# Rethinking Healthcare Quality and Prestige: Is This a Manager's Number One Problem?

**DOI:** 10.3389/fpubh.2022.863383

**Published:** 2022-03-29

**Authors:** Veronica Morales-Burton, Sofía A. Lopez-Ramirez

**Affiliations:** ^1^MSc Health Economics, Policy and Management, The London School of Economics and Political Science, London, United Kingdom; ^2^Department of Pediatrics, Fundación Cardioinfantil-Instituto de Cardiología, Bogotá, Colombia; ^3^School of Medicine and Health Sciences - Universidad del Rosario, Bogotá, Colombia

**Keywords:** healthcare quality assessment, accreditation (institutions), health services administration, organization and administration, health care economics and organizations

## Abstract

Healthcare institutions are organizations driven to provide medical assistance at a certain level of quality service and safety. To achieve the recognition of excellence, these entities can undergo accreditations and comparisons with other institutions of their kind through ranking systems in order to validate patient, organizational, and academic institutional standards. Usually, the goal is to obtain prestige and recognition as well as positive feedback toward the institution, motivating improvement. In this scenario, the manager's role is to communicate these results and propose strategies to maintain or increase healthcare quality. The following article discusses the fundamentals of the processes of accreditation and ranking systems, the importance of health managers on the complexity of these processes and on achieving an institution's goals and vision, but also intends to provide a critical view toward the desire for prestige a hospital envisions within the feedback when its biggest aim should be directed to improve in benefit of the patients and workforce conditions.

## Introduction

According to Peter Drucker, healthcare organizations are “the most complex form of human organization” (1), with a confluence of professions (physicians, administrators, pharmacists) and other stakeholders (patients, government), with diverse interests, which makes the managers' role in leading organizations a challenging one. How would one approach the job of managing the second-best healthcare organization in their country and fifth in the region according to a ranking system, besides retaining the international accreditation's golden seal? How should accreditations and rankings be approached?

## Accreditation Processes and Healthcare Standardization

Accreditation processes are part of healthcare systems, and since their creation in the early 1970s, they have been considered as indicators and important drivers to improve quality and safety in healthcare organizations. Accreditation systems have led to the creation of national and international programs, where external peer reviewers evaluate healthcare institutions and compare them to pre-established performance standards of care and quality, accepted at a global level (2). Currently, accreditation processes are being used internationally by policymakers and governmental entities to improve and standardize healthcare while pursuing transparency (2).

As an example, The Joint Commission International (JCI) is an organization whose mission is to improve safety and quality of care in the international community through education, advisory services, and international accreditation and certification processes (3). It works with healthcare providers, governments, public health agencies, academic institutions, and businesses to accomplish and maintain international standards of healthcare quality and patient safety (3). The healthcare accreditation criteria include international patient-centered standards, implying patient care safety goals, access and continuity of care, patient and family rights, assessment of patients, anesthetic and surgical interventions, medication management use, and patient and family education (3). It also includes Health Care Organization Standards such as quality improvement and patient safety, prevention and control of infection, governance, leadership, and direction, facility management and safety, staff qualifications and education, management of information, and academic medical center hospital standards (such as medical professional education, and human subject research programs) (3).

When an institution achieves accreditation criteria, it is given a symbolic “golden seal”, which is a national and internationally distinguished symbol that provides prestige to the organization, besides implying the challenge of maintaining standards of care, focusing on the organization's goals and vision (3). From a theoretical and ethical point of view, with no political, economic, or personal circumstances involved, what should a healthcare manager be aiming for if it is not this?

However, undergoing accreditation is costly and requires funds and time from healthcare organizations. Therefore, managers should perceive this as an organizational investment in quality and safety and, consequently, resources allocated. Healthcare policy should recognize this increase in costs and demand reports of results as trade-offs for reimbursement by stakeholders and contractors in healthcare. Thus, aiming to decrease future costs, reduce variation in medical practice, implement standardization of processes, and external validation of performance.

## Ranking Systems and the Dispute for Position

Besides accreditations, healthcare rankings are also in the scope of managers and hospital boards, where comparisons between healthcare organizations instigate a social process that gives them value within a general metric. In turn, this creates demands for information and dispute over categorization, far away from standardization, creating conflict within the public and staff, as their design does not usually align entirely with the institutional vision and strategy, or even with the broad and complex concept of healthcare, resulting in misinterpretation of performance if not treated with caution (4).

America Economía is one of the most renowned hospital ranking systems in Latin America. It positions accredited hospitals and compares them, considering seven dimensions in healthcare (5). The above mentioned are patient safety and dignity (indicators on patient security and hospital risk as well as transparency), workforce (leadership, government, and number of nurses and doctors), capacity (number of beds, number of specialties, number of surgeries performed, laboratory results), academic performance and impact (Journal publications), Efficiency (financial, medical results, quality), prestige, and patient experience (5).

Diverse hospital-standardization organizations, as well as ranking systems, have been used to compare healthcare within organizations. The reliability of the ranking hospitals' performance is assessed by determining the rank ability of multiple indicators that measure quality and execution throughout several areas of care. This includes providing information to patients, insurers, and policymakers and indirectly addressing transparency while impacting choice and reputation (6).

Rankings are not based solely on clinical outcomes, as they also include measurements on patient experience, safety, human resource, investment, and financial sustainability. Additionally, they review further aspects relevant to healthcare such as medical education and reputation, in which indicators are not examined as individual measurements with a particular outcome, but as composite measures used as an approach for safety and quality improvement. The comparison between organizations by rankings or national and international accreditation standards must be reviewed carefully by managers, whose job is to address the results while considering the organizations' visions and goals in an attempt to improve in standardization terms and to eliminate items that do not align with the institutions' objectives. Nevertheless, this is a challenging task given internal and external pressures as personal interests involving healthcare arise.

## The Role of the Healthcare Manager

As an alternative to the importance of the institution's position within a ranking system or accreditation program, healthcare managers should envision rankings and accreditations as an opportunity to improve, leading to a change of paradigms in which being on top, middle, or bottom should not be as relevant as improving results in the main areas of interest for each organization's mission and vision. Therefore, refinement and advances beyond previous weaknesses will improve scores, while the position on a list only reflects the speed of progress among institutions.

Understanding this involves changing patterns, pushing boundaries on existing categorization, and aligning strategy to what is being measured. Managers usually receive feedback and distribution of performance of the organization, and it is their responsibility to design and address an effective plan of action to at least maintain and hopefully exceed previous results (7). In such a complex scenario, strategy and leadership need to be collective and include every aspect of the organization; the four-frame scope (structural, political, symbolic, and human resources) is an adequate way of approaching the job (7).

Focusing strictly on the structural frame and point of view, organizations are created to achieve goals and objectives throughout a strategy which can be flexible depending on the circumstances. Roles, functions, and units are designed and described considering the organizations' goals and vision. Therefore, managers are expected to conduct their teams to accomplish those goals by adapting, communicating, and listening. Measuring outcomes, achieving positive indicators, and enhancing performance is part of the agenda. These are items used to compare organizations and discussions around them and should be used to learn and improve processes. Individual indicators such as mortality, readmission, infection rate, and composite indicators (mortality and readmission) have an immense impact on reputation, but so does patient preference. Having clear guidelines and pathways will result in efficiencies within the system, generating value, which is why the standardization of procedures' rules and policies needs to be well-defined.

Accreditation entities develop their programs and set their own standards from a structural and operational point of view in order to evaluate healthcare organizations to establish standards of care to improve quality and safety. Although each indicator is predominantly evaluated by assessing the organizational processes acquired to enhance them, without necessarily judging outcomes, a tendency toward measuring clinical and patient outcomes has been noted during the assessment. If applied in every accreditation process, this will guarantee that strategies are successful and encourage the workforce to maintain a higher level of care, despite the accreditation visit.

While the top positions in healthcare rankings may be achieved by top to bottom coordination, depending on their criteria, the complexity of accreditation processes in healthcare will need a more multifaceted structure that allows lateral coordination and communication to succeed (7, 8). On the human resource scope, and as described by Pink in his model of motivation for work in 2009, “motivation, purpose and mastery” play a determinant role in workforces (7). As in any other type of business, healthcare organizations need people with the appropriate skills to achieve goals, and individuals need organizations to respond to their needs, creating a relationship between them.

Strategies for improvement in accreditation processes cannot be implemented exclusively by governance or managers acting as individuals. It is a multidisciplinary task in which healthcare professionals from each level and area of the organization need to be involved, and it is the manager's job to motivate them to participate. Targeting standards in health is a process that requires to be constructed both individually and collectively to create a solid institutional setting that drives organizational culture. This facilitates efficiencies in the operation and gives individuals a role and purpose in their job, despite their thoughts about the experience, but with a desire for improvement. As discussed by Greenfield, involving, stimulating, and encouraging teams and individuals in the healthcare accreditation process is the strategy to lead quality and patient safety (9).

Moreover, performance feedback policies must be part of the manager's job. How these are designed and communicated within the organization and the teams will determine how individuals and groups react to performance as it increases the saliency of social comparison (10, 11). Performance can be measured by several instruments such as rankings. However, the way feedback and recognition are given to the organization plays an important role. Incentives motivate staff when implemented within the organization; they can be bonuses, promotions, and collective recognition or symbolic awards. Literature suggests that a positive effect is observed in those performing in top positions or bottom positions in the evaluation, being cautious and demoralizing in those middle-performance ranking workers (10).

On the other hand, external performance feedback, as the one received in accreditation processes, motivates the workforce to intensify their efforts and improve the quality of care. The symbolic incentive generated by different entities and the way the organization and its workers react is usually a positive driver and stimulant to the group. Depending on granulation, results may have a contrary effect and demotivate individuals, which is why fallouts and feedback demand appropriate communication to reach a positive behavioral effect on the organizations' workforce.

Relying on the political scope, the manager's task of maintaining an organization's status is important, and politics and power responding to accountability and responsibility play a determining role, as decisions need to be taken and resources allocated. As stated before, accreditation processes are collective exercises where the opportunity for coalition rises, and positive results attract investors as well as professionals that can offer value to the business.

As a manager, handling the decision of comparing the organization to others of their kind is a challenge that opens a competitive arena within healthcare organizations, allowing interaction with the government environment and society. An example of this is a national accreditation process. ICONTEC is a private non-profit organization recognized by the Colombian government as the National Standardization entity. It studies, adopts, and promotes technical standards in different economic and social activities, including healthcare and building credibility. It is also a way to compare healthcare organizations, aligning national standards and procedures within the country, which is considered the first step toward standardization in healthcare (12), thus creating a relationship between the government, patients, payers, and regulators within hospitals, which will result in financial sustainability while keeping quality of care and performance as well as reputation.

Although it may seem that each accreditation and standardization entity is strong enough to judge a healthcare organization, national, regional, and international entities have their advantages and thus, are attractive to assess individual indicators of an institution at their different extents. For instance, ICONTEC provides a guide in adhering to the National policies to standardize the country's health care. On a regional level, America Economía performs an annual comparison between hospitals in Latin America; this is an attractive ranking because of the similar social and demographic determinants of health within each country that can interfere with an institution's quality and capacity. Therefore, an institution can compare to similar others and annually acquire new goals and improvements to achieve higher positions each year in a similar environment. Finally, at a higher scope is Joint Commission International, which is appealing for institutions because it evaluates a healthcare institution in global standards regarding patient-centered safety, care, and experience and also takes into account workforce capacity, education, and academic quality. Therefore, their assessment and feedback encourage excellence at global scope, in the diverse aspects that shape a hospital comparable to ours, not only a center of medical attention but also a health training establishment. It motivates institutions to offer excellence beyond their limitations to be at the same level as others in the world.

In regard to the symbolic frame that shows hospitals as systems of myth and ceremony, where there needs to be meaning and value for what we do as healthcare workers, managers need to recognize and communicate to the organizations' workforce the negative and positive results in such a way to be able to achieve motivation, believe and find a meaning to everyday activities. JCI's goldenseal, for example, makes the organization proud of the results, motivates the workforce, and creates a culture based on standardized healthcare quality and patient safety. Therefore, it gives the organization a positive image to offer to the public, patients, and stakeholders, and encourages staff to perform better, bringing the organization together in a high-performance attitude.

## Conclusion

In conclusion, although rankings are a form of comparison that arises from external entities, managers and organizations usually have the responsibility to decide if they want to be compared with given criteria to others of their kind. At times driven by political pressures and sometimes to gain prestige, healthcare institutions can consider rankings as tools that bring advantages. However, rankings can also mislead the organizations as the categories involved will not always be aligned with the institutions' vision and goals, and quantitative results are not properly interpreted in terms of the sociology of quantification.

As a manager, having a clear image of the organizations' goals and vision, when faced with results on healthcare accreditation processes, will lead the organization through a quality and safety culture based on standardization, aiming to improve quality and patient care, as the standard of care, while challenging quantitative ranking results. This can be achieved by being cautious with interpretation and communication, and as stated by Espeland and Stevens, recognizing the sociology of quantification, the tendency of quantification to remake what it measures, channel social behavior, and learning the art of numerical expression as a tool for facing results (13).

“Numbers, like words, should be regarded as deeds: acts of communication, whose meaning and functions cannot be reduced to a narrow instrumentality, and which depend deeply on grammars and vocabularies developed through use” (13).

## Data Availability Statement

The original contributions presented in the study are included in the article/supplementary material, further inquiries can be directed to the corresponding author.

## Ethics Statement

All the research meets the ethical guidelines and has been approved by the Clinical Research Ethics Committee, Fundación Cardioinfantil-Instituto de Cardiología, Bogotá, Colombia. Approval number: IRB00007736.

## Author Contributions

VM-B contributed to the first draft of the manuscript. SL-R contributed to the summary and the editing and reviewal of the manuscript. All authors contributed to the conceptualization of this viewpoint, made a substantial contribution to the design of the work, contributed to the bibliography, the refinement of the final version, and have approved and accepted responsibility for the entire content of the final manuscript.

## Conflict of Interest

The authors declare that the research was conducted in the absence of any commercial or financial relationships that could be construed as a potential conflict of interest.

## Publisher's Note

All claims expressed in this article are solely those of the authors and do not necessarily represent those of their affiliated organizations, or those of the publisher, the editors and the reviewers. Any product that may be evaluated in this article, or claim that may be made by its manufacturer, is not guaranteed or endorsed by the publisher.
